# Schwartz Centre Rounds: qualitative exploration of panel members’ experiences within a forensic mental health service

**DOI:** 10.1192/bjb.2021.132

**Published:** 2023-04

**Authors:** Emma Groves, Rachel Collinson, Amy Hegarty, Elshiema Hamad, Tyler-Dee Asadi, Abebe Ejara, Lubna Kudsia, Shivali Shah

**Affiliations:** 1 Tees, Esk and Wear Valleys NHS Foundation Trust, UK; 2 Secure Inpatient Services, Roseberry Park Hospital, UK

**Keywords:** Schwartz Centre Rounds, reflective practice, teamworking, resilience, forensic mental health services

## Abstract

Schwartz Centre Rounds (SCRs) provide a structured forum for staff from all disciplines to meet and discuss the difficult emotional and social challenges that arise in caring for patients. Research into the implementation of SCRs has shown that staff who attend report increased insight into the emotional and social aspects of care; greater understanding of the roles of their colleagues; improved teamworking and decreased feelings of isolation and stress. However, little research has explored the implementation of SCRs within forensic settings, and no research has focused solely on the experiences of panel members. Three focus groups were facilitated with participants who had participated in a SCR panel within a forensic mental health service. Semi-structured interviews were carried out, audio-recorded and subsequently transcribed. Interpretive phenomenological analysis was utilised to analyse the transcripts, and four key themes were identified. These themes were: feeling vulnerable, the importance of validation, exposure to intense emotional experiences and improved understanding and connection. We conclude that SCRs can be an emotionally challenging but rewarding experience, with the potential to enhance teamworking and general well-being. Limitations and recommendations for future research are also discussed.

High levels of stress and burnout are highly prevalent amongst front-line staff in mental health settings, and this is particularly evident within forensic psychiatric services. Whittington and Richter^1^ found that being repeatedly exposed to violence and aggression was a key factor in high rates of occupational stress and burnout. Yet, despite these pressures, staff are responsible for providing care to some of society's most complex individuals. Thus, the importance of providing adequate support for staff members in such environments has been highlighted, in particular the need to develop an awareness of staff vulnerabilities, as well as fostering a culture of speaking openly about feelings.^2^ In addition, space for reflection is seen to be vital for front-line staff to be able to take care of themselves, develop self-awareness and subsequently provide better patient care.^3^

## Background

Schwartz Centre Rounds (SCRs) provide a monthly forum for multidisciplinary teams to explore together the emotional and social challenges that arise in caring for patients. The purpose is not to discuss clinical aspects of patient care or solve problems. SCRs were developed in the late 1990s in the USA, by a young lawyer named Kenneth Schwartz, who received care for terminal lung cancer. Kenneth was struck by the simple acts of kindness that he experienced in his last few weeks and so, before his death, he set up the Schwartz Centre in Boston to help develop compassion in healthcare. In 2009, SCRs were first replicated in the UK by the Point of Care Programme at The King's Fund, and they continue to be implemented by The Point of Care Foundation.

SCRs follow a standard model that is replicated across all settings and normally take place once a month for 1 hour, with catering provided. They consist of a panel of three to four professionals from different backgrounds (clinical and non-clinical), who individually tell their stories for the first 15–20 minutes. Afterwards, there are two trained facilitators who lead an open discussion, guiding reflection and steering away from problem-solving for the remainder of the SCR. Following the SCR, all attendees are asked to complete an evaluation form.

Lown and Manning^4^ explored the impact of SCRs by conducting retrospective surveys of attendees at six sites who had implemented SCRs. Most of the retrospective survey respondents indicated that attendance enhanced their likelihood of attending to psychosocial and emotional aspects of care, and enhanced their beliefs about the importance of empathy. Respondents reported improved teamworking, including heightened appreciation of the roles and contributions of colleagues. There were significant decreases in perceived stress and improvements in the ability to cope with the psychosocial demands of care. The authors concluded that SCRs may foster enhanced communication, teamwork and provide support. Findings also showed that the impact on measured outcomes increased with the number of SCRs attended.

A mixed-method approach was utilised by Chadwick et al^5^ to evaluate the impact of SCR on the staff of a large acute general hospital over a 3-year period. Evaluation data was collected routinely from all staff attending over this period, and analysed quantitatively and qualitatively. The findings showed a very positive response to all aspects of the SCR by staff who attended. The most highly rated statement was: ‘I have gained insight into how others think/feel in caring for patients’. This was reinforced by the qualitative analysis in which the primary theme was found to be ‘insight’. There were no significant differences between disciplines/staff groups, indicating that all staff members, whether clinical or non-clinical, responded to the SCR equally positively. They concluded that SCRs were highly valued by staff from all disciplines, and by managers and other non-clinicians as well as clinicians. Furthermore, SCRs appear to have the potential to increase understanding between different staff members, and thus reduce isolation and provide support.

To understand the individual experiences of those who were involved in implementing SCRs, Goodrich^6^ undertook 41 interviews across two National Health Service (NHS) hospitals providing acute care. SCRs were perceived by participants as a source of support and that their benefit may translate into benefits for patients and teamworking. In addition, some felt that that SCRs had the potential to effect change in the hospital culture. They determined that SCRs appeared to transfer successfully from the USA to the UK, and there was evidence that they were having a similarly positive effect; they added that further research is required to substantiate these findings.

To date, no research has explored the implementation of SCRs within forensic settings. In light of the challenges that face front-line staff in forensic mental health services, the current research aimed to explore the experiences of staff who had been involved in telling their story on an SCR panel.

## Method

### Ethics and consent

The authors assert that all procedures contributing to this work comply with the ethical standards of the relevant national and institutional committees on human experimentation and with the Helsinki Declaration of 1975, as revised in 2008. All procedures involving human patients were approved by the Health Research Authority and Health and Care Research Wales. The Integrated Research Application System project identifier is 241483.

Informed written consent was obtained from all participants; participants were given all necessary information regarding the research and offered the opportunity to ask questions before signing the consent forms.

### Participants

The research team recruited participants via the following methods: discussions and expressions of interests from previous panel members within SCR steering group meetings; and email invites were sent to staff members who had been panel members (names were sourced from a confidential database within the service).

Eight participants (clinical and non-clinical) were recruited to the study in total from a forensic mental health service in North-East England. Participants included one senior service manager, one speech and language therapist, one support worker, two nurses, one domestic assistant, and two medical secretaries. Seven of the participants were female and one was male. Each participant had experience of being a panel member on an SCR within the past 2 years.

### Data collection

Three focus groups were facilitated by members of the research team and involved semi-structured interviews, averaging 1 hour in duration. All focus groups were audio-recorded separately via a service-encrypted voice recorder. Each focus group was then transcribed and stored on a password-protected NHS computer. All documents were password-protected and only specific members of the research team had access.

The focus groups were conducted with a researcher:participant ratio of 2:2 or 2:3, and took place on different dates with different researchers interviewing. Participants were asked each question individually, in turn, and were given the opportunity to elaborate on their experiences with further questioning by the researchers. The focus groups were facilitated to ensure individual responses to each question were captured, to explore each individual experience – the very essence of interpretive phenomenological analysis, which was used to analyse the data. Exploration of participants’ shared experience within the focus groups also allowed them to make sense of their experiences through lengthy discussions, disagreements and agreements with one another, which further enriched the results. The transcripts of the recordings clearly indicated the account of each participant, so that these could be analysed in depth accordingly. The researchers therefore concluded that the use of focus groups allowed for exploration of individual and shared experiences, consequently providing a rich and in-depth data-set.

### Data analysis

The chosen method of data analysis was interpretive phenomenological analysis, and this followed the six stages of data analysis set out by Smith et al.^7^ Analysis was conducted and triangulated by five members of the research team (E.C, R.C, E.H, T.-D.A. and A.E). Stage 1 consisted of reading and re-reading. By reading and re-reading the transcripts of the interviews, the researchers were able to immerse themselves and areas of rich data were identified. Stage 2 consisted of initial noting. Researchers began to note things that mattered to the participant and what it meant for the participant to have these experiences. Stage 3 consisted of developing emerging themes. Researchers looked for connections and patterns within the notes taken from stage 2. Themes were noted and the detail was reduced without losing the complexity of the data. Stage 4 consisted of searching for connections across emergent themes. At this point the researchers attempted to map out the themes to see how they fit together, how they differed and the function that they may have served. Stage 5 consisted of moving on to the next case. It was important to consider each transcript as an individual and unique transcript. Finally, stage 6 consisted of looking for patterns across cases. This was the stage whereby researchers looked for similarities between the themes for each transcript. By clustering themes together, researchers were left with a number of superordinate themes.

The final element of the data analysis was to develop a narrative that evidenced excerpts from the transcripts to produce a detailed commentary.

## Results

Four superordinate themes were identified through the data analysis: ‘feeling vulnerable’, ‘the importance of validation, ‘exposure to intense emotional experiences’ and ‘improved understanding and connection’. Each theme was evidenced with data from at least half of the participants corresponding to recommendations from Smith et al.^7^

### Feeling vulnerable

Panel members reported feeling apprehensive about sharing their experiences, and this apprehension was interpreted as stemming from a fear for of saying something that was incongruent with others’ beliefs or experiences. Participant G describes a concern that others may not be interested in what he had to say:

‘You just wonder if the people who are sitting there are actually going to be interested in this, are they going to start judging me?’ Participant G.

‘I thought no one would take my story seriously.’ Participant A.

The concept of vulnerability was juxtaposed with the idea that professionals in the organisation come to work wearing a mask and, by taking part in an SCR, they were in some way venturing out of their comfort zone. This was evidenced by participant S, who reflected on how she usually prefers to present herself as able to cope and that by taking part in the SCR, she may be showing a more vulnerable side to herself.

‘I was anxious at first as I thought I was lowering my guard a bit, because you always put on this face of I can do anything, I can cope with everything.’ Participant S.

An additional sense of vulnerability came from an awareness of senior members of staff who were sometimes present at SCRs. For those who were not in senior positions in the organisation, this appeared to be somewhat a barrier and led to individuals questioning whether they deserved to be present at SCRs.

‘There was that initial feeling of oh my goodness there are Consultants in the audience, I almost felt like I didn't deserve to be there because I don't directly deal with patients.’ Participant A.

### The importance of validation

Validation from others was extremely important to all panel members, and this appeared to have a positive effect. The importance of hearing others share similar experiences was interpreted as a protective factor against feelings of isolation:

‘It was nice to hear other people's experiences, we were similar and we are not alone.’ Participant J.

For participant R, hearing other's stories that were similar to her own, contributed to a sense of calmness:

‘They were chipping in and agreeing, that put me at ease.’ Participant R.

For participant C, the practice run normalised her experiences, as hearing others tell similar stories allowed her to reframe her beliefs about not having enough time for her clinical duties:

‘I wasn't saying anything out of the ordinary and of course they will understand why we run out of time as clinicians. That was a useful practice run I think.’ Participant C.

### Exposure to intense emotional experiences

The SCR often triggered intense emotional experiences for all participants, and for participant H this seemed unexpected:

‘I felt that this is what it must be like to have a personality disorder, one minute I felt such a relief and then the next minute, oh my god, I can't keep my emotions in check!’ Participant H.

For some, connecting to emotions that are usually bottled up was seen as a positive as it is not an experience that they often have an outlet for:

‘I felt really emotional on one of the Schwartz Rounds I did and it was really helpful to hear other people's experiences because I kept a lot of things bottled up.’ Participant R.

However, this exposure to emotions in the SCR was not necessarily welcomed by panel members, as there seemed to be a belief that expressing emotions in front of colleagues is undesired. This may explain and underpin the perceived feelings of vulnerability discussed above.

‘I think emotions can run away with you and I noticed that one of the other people were crying and I know that during it I was close to tears, so from that point of view it was quite a negative thing.’ Participant H.

There seemed to be an understanding that SCRs were unique in the way that they place people in touch with their emotions whilst in the work environment. It was apparent that there was a shared belief that emotions are often ‘bottled up’ or supressed in some way when at work, and so connecting with these emotions feels unnerving and potentially exposing.

### Improved understanding and connection

Despite the fear of exposing oneself and connecting with their emotions, participants reported how SCRs can often foster connection and understanding among colleagues.

‘I am a huge advocate of them, I think they are really, really helpful. They are very good to give you an understanding of our colleagues who you don't always see or hear from.’ Participant J.

‘It did probably make me think of other people at work a little more.’ Participant B.

For some, it was an opportunity to share their experiences of their role and improve understanding, as well as perhaps also to gain a sense of validation that they provide a valuable contribution to the service:

‘In my role, nobody has an understanding of what I do so it was an opportunity for other people to get an insight into what I was doing.’ Participant S.

‘This gives you an ideal opportunity to find out and think oh so that is what you do!’ Participant G.

For some, sharing a story and expressing emotions brought about a sense of affiliation and connection to others. Perhaps because allowing oneself to show their human side is seen as extremely exposing, the personal impact is significant, and this was reflected by participant S:

‘It did feel like a personal achievement for me to do it and I felt part of something.’ Participant S.

Participants’ responses reflected a journey from intense nerves and apprehension through to overcoming fear of judgement, resulting in increased understanding of others and a chance to connect with colleagues on an emotional level.

## Discussion

Previous research into implementation of SCRs has shown that staff members who attend report a greater understanding of the roles of their colleagues and improved teamworking. The findings of the current research support this and highlight the importance of being able to educate others about their experiences, as well as learn about the roles of their colleagues.

The qualitative analysis revealed four superordinate themes, some of which echoed findings of previous research. First, the theme entitled ‘the importance of validation’ highlighted the importance of hearing others share similar experiences, and this experience was interpreted as a protective factor against feelings of isolation. Dewall and Bushman^8^ found that SCRs were perceived by participants as a source of support, and that their benefit may translate into benefits for patients and teamworking. When considering the key tenets of social identity theory,^9^ the societal desire for validation can be explained by an evolutionary need to be part of a group. It is these groups that contribute to individuals’ sense of social identity, pride and self-esteem, and thus to be rejected from certain social groups represents great threat and causes intense anxiety. This may explain why in SCRs, expressing honest and open views about one's personal experiences risks incongruence and perhaps rejection from colleagues, in other words, the ‘in group’. This potentially explains why the analysis uncovered an additional superordinate theme of ‘feeling vulnerable’.

Lown and Manning^4^ explored the impact of SCRs, focusing on changes in attendees’ self-reported behaviours and beliefs about patient care, sense of teamwork, stress and personal support. They found that respondents reported better teamwork, including heightened appreciation of the roles and contributions of colleagues. They also stated that SCRs may foster enhanced communication and teamwork. This finding is echoed within the superordinate theme found within this evaluation entitled ‘improved understanding and connection’.

Findings reported by Chadwick et al^5^ evidenced a very positive response to all aspects of the SCR. The most highly rated statement on the evaluation questionnaire was: ‘I have gained insight into how others think/feel in caring for patients’. They concluded that SCRs appear to have the potential to increase understanding between different staff members, and there was no time for day-to-day conversations that help staff to understand each other. They also felt that comments provided within their research highlighted the ‘emotional content’ of SCRs, and concerns that SCRs may elicit emotions that cannot be contained and thus cause harm. These concerns were echoed within the current research, and a sense of vulnerability was communicated by participants.

As demonstrated in the findings, the current research adds greater depth to the results of previous studies. In particular, highlighting that SCRs are equally useful in forensic services, which has not been explored previously. The implementation of SCRs in forensic settings can add value to the service and should be encouraged alongside the various support networks available for staff. This research highlights the universal benefit of SCRs in mental health settings, and the researchers recommended that future research explores the implementation of SCRs across a wide variety of mental health settings.

### Limitations

It must also be noted that the results presented in this research were based on the subjective interpretations of the researchers. Reflective diaries may have allowed transparency in the interpretative process and reduced subjectivity. The qualitative analysis was based on focus group interviews. It is acknowledged that there is a possibility that, because of being in a group setting, participants may not have fully expressed their views or may have provided answers in line with those of their peers. Within this research, two to three participants took part in each focus group. If replicated, a higher number of participants is recommended, in line with current research that suggests communication is positively influenced by smaller group sizes of between four and six participants in size.^10^

### Implications

Participants reported feeling apprehensive during SCRs, and this appeared to stem from a fear of saying something that differed from the beliefs or experiences of others, leading to the superordinate theme of ‘feeling vulnerable’. An additional sense of vulnerability came from an awareness of senior members of staff who were sometimes present at SCRs. This led to individuals questioning whether they deserved to be present at SCRs. This highlights the need for SCRs to be promoted as a safe space for all to share their experiences openly and honestly, without fear of the presence of senior members of staff. For SCRs to be successful, reducing any sense of hierarchy is vital.

The analysis revealed that participants did not often volunteer to be on the panel, and were usually asked and approached by colleagues to share their stories. This implies a need to foster an approach whereby SCRs are widely promoted within services, to improve understanding and increase the likelihood of participants volunteering. If participants were to volunteer, this may increase feelings of empowerment and control over the participant and potentially reduce feelings of vulnerability.

Participants spoke about valuing the preparation undertaken before the SCR taking place in order to share their stories, and this remains an integral part of being a panel member. Some also felt that a debrief following the SCR would be beneficial, and provide a space to reflect on their emotional experience and share any subsequent concerns. Research by Chadwick et al^5^ also acknowledged this and that attendees of the SCR are purposely not offered further support outside of the rounds for fear that this implies that sharing emotions is not encouraged. However, this does raise important considerations regarding support given following the SCR, and perhaps this needs to take place on an individualised basis.

### Future research

It remains apparent that further research is needed to evaluate the impact of SCRs and their utility within a forensic context. Further qualitative analysis is recommended to understand the individual experiences of SCRs for attendees. This would potentially uncover the more subtle and nuanced processes that underpin the apparent success of SCRs. Results show that SCRs can lead to changes in practice and improved understanding of the roles of colleagues; however, further research is required to find out how this affects the practice of those that attend SCRs.

To summarise, four key themes were identified within the current research. These themes highlighted a journey that SCR panel members embark upon when they undertake this role. The fear of expressing emotion and vulnerability in front of colleagues was apparent and underpinned intense nervousness and apprehension. However, SCRs appear to provide an opportunity to experience validation, and therefore overcome the fear of judgement when expressing emotional experiences within the context of work. Overall, both participants and researchers concluded that SCRs have the potential to increase understanding of others and provide a chance to connect with colleagues on an emotional level, thus enhancing relationships and teamworking. Further research is needed into the experiences of attendees, to understand the impact that SCRs have on their own emotional well-being and subsequent clinical practice. It is these individual stories and narratives that have the potential to shape and guide the development of future SCRs.

## Data Availability

The data that support the findings of this study are available on request from the corresponding author, E.G. The data are not publicly available due to their containing information that could compromise the privacy of research participants.

## About the authors

**Dr Emma Groves** is a highly specialist clinical psychologist with Secure Inpatient Services at Roseberry Park Hospital, at Tees, Esk and Wear Valleys NHS Foundation Trust, UK. **Dr Rachel Collinson** is a highly specialist clinical psychologist with Secure Inpatient Services at Roseberry Park Hospita,l at Tees, Esk and Wear Valleys NHS Foundation Trust, UK. **Dr Amy Hegarty** is a senior registrar in forensic psychiatry with Secure Inpatient Services at Roseberry Park Hospital, at Tees, Esk and Wear Valleys NHS Foundation Trust, UK. **Dr Elshiema Hamad** is a senior registrar in forensic psychiatry with Secure Inpatient Services at Roseberry Park Hospital, at Tees, Esk and Wear Valleys NHS Foundation Trust, UK. **Tyler-Dee Asadi** is a higher assistant psychologist with Secure Inpatient Services at Roseberry Park Hospital, at Tees, Esk and Wear Valleys NHS Foundation Trust, UK. **Dr Abebe Ejara** is a consultant psychiatrist with Secure Inpatient Services at Roseberry Park Hospital, at Tees, Esk and Wear Valleys NHS Foundation Trust, UK. **Lubna Kudsia** is a higher assistant psychologist with Secure Inpatient Services at Roseberry Park Hospital, at Tees, Esk and Wear Valleys NHS Foundation Trust, UK. **Dr Shivali Shah** is a senior registrar in forensic psychiatry with Secure Inpatient Services at Roseberry Park Hospital, at Tees, Esk and Wear Valleys NHS Foundation Trust.
